# Consequences of marine barriers for genetic diversity of the coral‐specialist yellowbar angelfish from the Northwestern Indian Ocean

**DOI:** 10.1002/ece3.5622

**Published:** 2019-09-04

**Authors:** Felipe Torquato, Pedro Range, Radhouane Ben‐Hamadou, Eva E. Sigsgaard, Philip F. Thomsen, Rodrigo Riera, Michael L. Berumen, John A. Burt, David A. Feary, Alyssa Marshell, Daniele D'Agostino, Joseph D. DiBattista, Peter R. Møller

**Affiliations:** ^1^ Section for Evolutionary Genomics Natural History Museum of Denmark University of Copenhagen Copenhagen Denmark; ^2^ Environmental Science Center Qatar University Doha Qatar; ^3^ Department Biological and Environmental Science College of Arts and Sciences Qatar University Doha Qatar; ^4^ Department of Bioscience University of Aarhus Aarhus Denmark; ^5^ Departamento de Ecología Facultad de Ciencias Universidad Católica de la Santísima Concepción Concepción Chile; ^6^ Division of Biological and Environmental Sciences and Engineering Red Sea Research Center King Abdullah University of Science and Technology Thuwal Saudi Arabia; ^7^ Center for Genomics and Systems Biology New York University Abu Dhabi Abu Dhabi UAE; ^8^ MRAG Ltd London UK; ^9^ Marine Ecology Lab Oman Department of Marine Science and Fisheries College of Agriculture and Marine Science Sultan Qaboos University Muscat Oman; ^10^ School of Life Sciences University of Nottingham Nottingham UK; ^11^ School of Molecular and Life Sciences Curtin University Perth WA Australia; ^12^ Australian Museum Research Institute Australian Museum Sydney NSW Australia

**Keywords:** connectivity, coral reef fish, marine biogeography, phylogeography, *Pomacanthus maculosus*, seascape genomics

## Abstract

Ocean circulation, geological history, geographic distance, and seascape heterogeneity play an important role in phylogeography of coral‐dependent fishes. Here, we investigate potential genetic population structure within the yellowbar angelfish (*Pomacanthus maculosus*) across the Northwestern Indian Ocean (NIO). We then discuss our results with respect to the above abiotic features in order to understand the contemporary distribution of genetic diversity of the species. To do so, restriction site‐associated DNA sequencing (RAD‐seq) was utilized to carry out population genetic analyses on *P. maculosus* sampled throughout the species’ distributional range. First, genetic data were correlated to geographic and environmental distances, and tested for isolation‐by‐distance and isolation‐by‐environment, respectively, by applying the Mantel test. Secondly, we used distance‐based and model‐based methods for clustering genetic data. Our results suggest the presence of two putative barriers to dispersal; one off the southern coast of the Arabian Peninsula and the other off northern Somalia, which together create three genetic subdivisions of *P. maculosus* within the NIO. Around the Arabian Peninsula, one genetic cluster was associated with the Red Sea and the adjacent Gulf of Aden in the west, and another cluster was associated with the Arabian Gulf and the Sea of Oman in the east. Individuals sampled in Kenya represented a third genetic cluster. The geographic locations of genetic discontinuities observed between genetic subdivisions coincide with the presence of substantial upwelling systems, as well as habitat discontinuity. Our findings shed light on the origin and maintenance of genetic patterns in a common coral reef fish inhabiting the NIO, and reinforce the hypothesis that the evolution of marine fish species in this region has likely been shaped by multiple vicariance events.

## Graphical abstract

Metapopulation genetics of coral reef fish. Phylogeography Northwestern Indian Ocean.

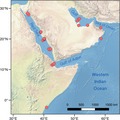

## 1 INTRODUCTION

Coral‐dependent fishes occupy relatively discrete patches of habitat as adults that can be separated by areas of unsuitable habitat ranging in scale from few meters to thousands of kilometers (Morrison & Sandin, 2011; Planes, 2002). Patterns of distribution and interchange across such habitat patches will be predominantly associated with species pelagic life stage, as subadults and adults present species‐specific levels of homing behavior (Mumby, 2006). Nearly all demersal marine teleosts have a bipartite life cycle in which adults produce tiny propagules (i.e., ichthyoplankton) that undergo a pelagic larval phase, potentially migrating between patches before settling and metamorphosing into juveniles (Leis, 2006). Therefore, this life stage represents the first and most predominant opportunity for dispersal, and consequently where interpopulation connectivity may likely occur (Cowen, Lwiza, Sponaugle, Paris, & Olson, 2000; Cowen, Paris, & Srinivasan, 2006).

The inter‐patch distance and the movement capacity of the larvae may strongly impact population structure by the accumulation of local genetic differences through space. For example, if the distance of larval migration is much smaller than the species range, the population will be structured such that genetic and geographic distance between populations is positively correlated, resulting in patterns of isolation‐by‐distance (IBD; Wright, 1943). On the other hand, independent of geographic distance, the population may be structured due to the influence of seascape heterogeneity on gene flow and population connectivity. In this scenario, genetic differentiation increases with environmental differences (i.e., Isolation‐by‐environment; IBE) due to natural selection against immigrants, sexual selection against immigrants, reduced hybrid fitness, and/or biased dispersal (Wang & Bradburb, 2014).

In contrast to these thoughts, the high fecundity of marine fishes combined with prevailing oceanographic currents and extended pelagic larval durations (PLD) would be expected to result in substantial larval dispersion potential, in which adult populations are variously interconnected (i.e., panmictic) and, therefore, no correlation exists between geographic and genetic distances. Biogeographic studies of marine fishes have demonstrated the existence of numerous endemic species around isolated oceanic islands and in peripheral areas (e.g., Red Sea), suggesting that larval retention can occur in a myriad of ways even in short spatial scales. Indeed, in addition to distances and heterogeneous seascapes, historical and oceanographic barriers are also thought to be important factors affecting larval dispersal and consequently population structure (Bowen et al., 2016; Bowen, Rocha, Toonen, & Karl, 2013). For example, the biogeographic patterns of adult reef fishes inhabiting the Northwestern Indian Ocean (NIO) indicate no single, repeated, or uniform explanation to larval retention. Instead high variance in contemporary species distributions is likely the outcome of a number of vicariance events that involve historical and contemporary barriers (Berumen, DiBattista, & Rocha, 2017).

In the NIO, historical sea level changes within the Red Sea and Arabian Gulf associated predominantly with Pleistocene epoch glacial cycles have impacted the historical availability of habitat, direction, or magnitude of oceanographic currents, and abiotic conditions within both regions, substantially impacting on current levels of biodiversity (Riegl & Purkis, 2012). Water exchange between the Red Sea and the rest of the Indian Ocean, through the Strait of Bab al Mandab, has been repeatedly restricted during Pleistocene glacial cycles (Stevens et al., 2014) when sea level was lowered by ~130 m (Clark et al., 2009). In comparison, the Arabian Gulf reached its present levels just 6–9 K years ago, with the entire seabed therefore exposed during the Pleistocene period (Vaughan, Al‐Mansoori, & Burt, 2019; Lokier et al., 2015).

In turn, contemporary dispersal barriers for coral reef fishes within the NIO are mainly structured by southwest monsoonal activity, resulting in seasonal cold‐water upwelling events, which can hinder planktonic dispersal of species intolerant to low temperatures (Hoeksema, 2007). In addition, such oceanographic event have also led to large areas of unsuitable larval settlement habitat in the upwelling zones, potentially isolating populations by restricting stepping‐stone connectivity and thereby increasing the chance for vicariant splits (Burt et al., 2016; Priest et al., 2016).

The geographic position and consequences of such barriers on reef fish distribution within the NIO have been previously investigated, with such works relying predominantly on species occurrence data (Burt et al., 2011; DiBattista, Choat, et al., 2016; DiBattista, Roberts, et al., 2016; Kemp, 1998, 2000; Klausewitz, 1972, 1989). Increasing work about these barriers has now focused on surveying the population genetic structure of conspecifics throughout their range, as genetic discontinuities may provide insight into past and present barriers and allow historical inferences on dispersal (Berumen et al., 2017). Despite these endeavors, there is still little understanding of the mechanisms underlying the distribution of biodiversity of coral reef fishes within the NIO (see, Bowen et al., 2013; DiBattista, Choat, et al., 2016; DiBattista, Roberts, et al., 2016).

In this paper, we investigate population genetic structure of the yellowbar angelfish *Pomacanthus maculosus* (Pomacanthidae; Forsskål 1775), a common coral‐dependent fish found throughout the NIO. We first examine whether intraspecific genetic variation has been driven by either IBD or IBE, and if not, we examine whether genetic variation within *P. maculosus* aligns with contemporary species distribution patterns by investigating the role of the putative marine barriers in the NIO.

## 2 MATERIAL AND METHODS

### 2.1 Study area

**Figure 1:**
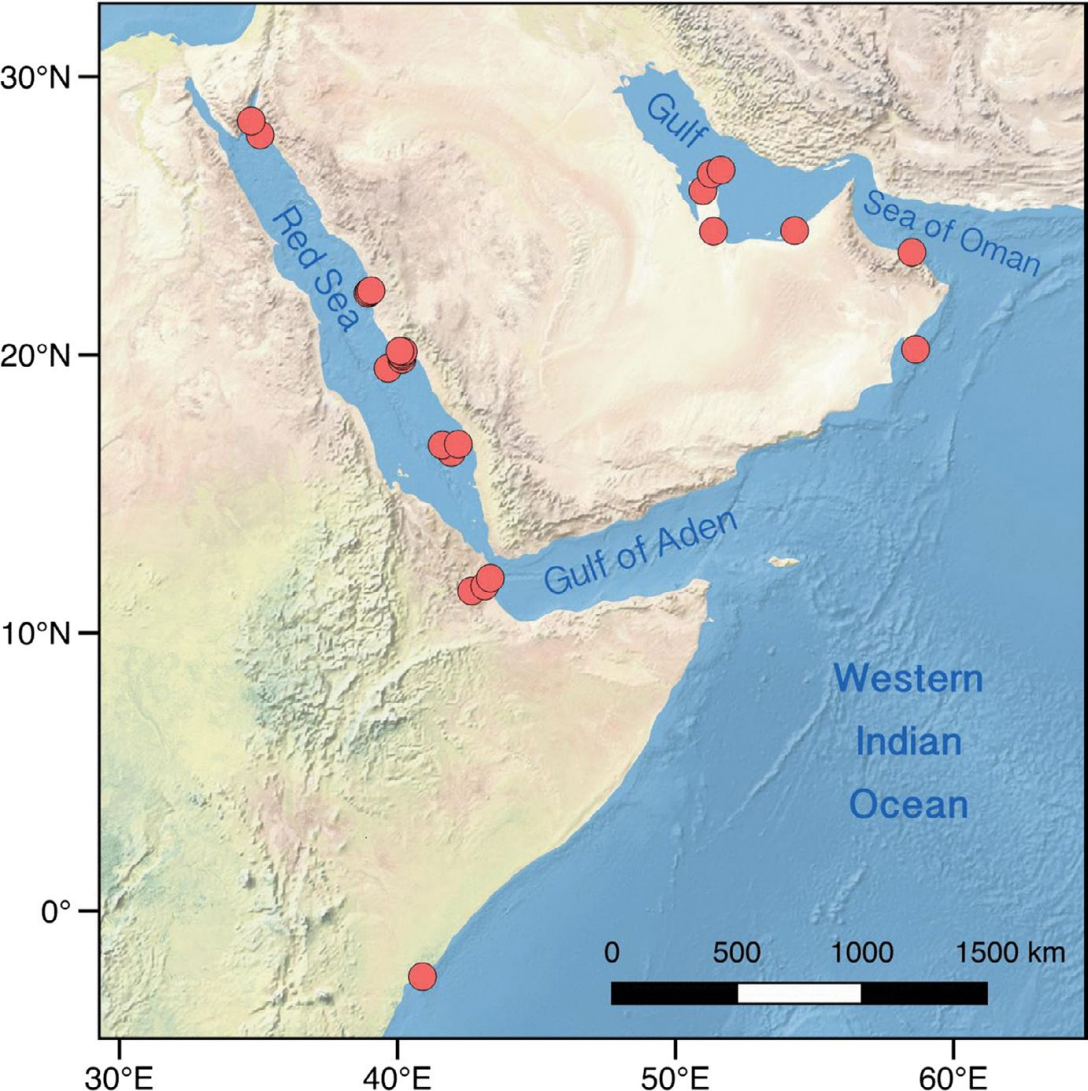
Locations of the 27 sampling sites for *Pomacanthus maculosus* across the Western Indian Ocean

The study region comprised the Northwestern Indian Ocean (NIO): northeast of Kenya on the eastern African coastline (hereafter referred to as Kenya), and the seas surrounding the Arabian Peninsula, that is the Red Sea, the Gulf of Aden, the Arabian Sea, the Sea of Oman, and the Gulf (also known as the Arabian Gulf or Persian Gulf; Figure 1). Coral reef habitat persists across the NIO, despite substantial gradients in environmental features. In the Arabian Gulf, for example, sea surface temperature (SST) ranges from 12°C in winter to over 36°C in summer, and salinity is often >45 (Reynolds, 1993). In the Red Sea, salinity can reach 42.5 (Medio et al., 2000), and SST can rise to values of between 36 and 38°C in the south, while temperatures as low as 10°C have been recorded in the Gulf of Suez in the north. In contrast, both the Arabian Sea and the ocean off Kenya exhibit moderate salinity (36–37) and relatively cool temperatures (20–26°C and 25–29°C, respectively; Burt et al., 2011; Kayanne et al., 2006).

The NIO exhibits more dramatic seasonal variations in abiotic parameters than the rest of the Indian Ocean (Benny, 2002). The water mass distribution and upper ocean circulation in the NIO changes in response to biannual wind stress reversals, creating seasonality in oceanographic conditions (Shetye & Shenoi, 1994). During winter (November–March), the monsoon wind blows from the northeast, away from the Asian continent, and the ocean surface circulation in the Arabian Sea is approximately counter‐clockwise. This pattern is reversed during the summer monsoon (May–September) when the wind blows strongly from the southwest, and the circulation in the Arabian Sea is clockwise (see Cutler & Swallow, 1984).

### 2.2 Sampling design and DNA extraction

The sampling scheme was designed to maximize the geographic and environmental distances covered, so as to best investigate the importance of these factors for population genetic variation within *P. maculosus*. Therefore, a total of 151 tissues samples (fin clips) were collected from 27 locations that together covered an extensive part of the distributional range of the species, including the Red Sea, the Gulf of Aden, the Sea of Oman, the Arabian Gulf and Kenya (Figure 1 and Table 1). Fin clips were preserved in 96% ethanol and kept at −20°C until DNA extraction. High‐molecular‐weight genomic DNA was isolated in a final elution volume of 100 μl following the manufacturer's instructions using either the Qiagen DNeasy^®^ Blood & Tissue Kit or the KingFisher Cell and Tissue DNA Kit. DNA concentrations in the extracts were measured using the Qubit 2.0 dsDNA BR Assay Kit (Invitrogen^™^) Fluorometer and checked for high‐molecular‐weight bands on a 1% agarose gel.

**Table 1:** Geographic locations of sampling sites and sample sizes for *P. maculosus*

Region	Country	Sampling site	Coordinates	Number of samples
Gulf of Aden	Djibouti	Maskali	N 11°41′, E 43°08′	13
Gulf of Aden	Djibouti	Bay de Ghoubbet	N 11°30′, E 42°40′	4
Red Sea	Saudi Arabia	Farasan Island (North of Shuma, Mahama, Abulad Island)	N 16°45′, E 41°36′	11
Red Sea	Saudi Arabia	Jazirat Burcan	N 27°54′, E 35°03′	1
Red Sea	Saudi Arabia	Gulf of Aqaba	N 28°24′, E 34°44′	2
Red Sea	Saudi Arabia	Dolphin Lagoon	N 19°31′, E 39°39′	2
Red Sea	Saudi Arabia	North Abu Latt, Saut, S. Sulaym	N 19°57′ E 40°09′	9
Red Sea	Saudi Arabia	Al‐Fahal, Om Al Balak, Al Wusul	N 22°13′, E 38°57′	10
Red Sea	Saudi Arabia	Middle Reef, Shib Habil, Manila Bay	N 20°07′ E 40°12′	10
Red Sea	Saudi Arabia	Abu Shosha	N 22°18′ E 39°02′	6
Sea of Oman	Oman	Fahal Island	N 23°40′ E 58°30′	13
Arabian Sea	Oman	Masirah Island	N 20°09′, E 58°38′	3
Arabian Gulf	Abu Dhabi	Saadiyat	N 24°31′, E 54°26′	24
Arabian Gulf	Qatar	Umm Al‐Arshan, Al Rayan Reef, Al Zubara	N 26°31′, E 50°17′	34
Africa	Kenya	Lamu	N 2°16′ E 40°54′	9

### 2.3 Genotyping and de novo assembly of RAD tags

RAD tag libraries were constructed by Floragenex from two 96‐well plates, following the protocol outlined by Baird et al. (2008), Hohenlohe et al. (2010) and Etter (2011). In brief, high‐molecular‐weight genomic DNA was digested into small fragments with a high fidelity SbfI restriction enzyme, and an adapter (P1) containing a matching sticky‐end TGCAGG and in‐line barcode sequence was ligated to the fragment's overhanging ends. Tagged restriction fragments from all individuals were then pooled (multiplexed), randomly sheared and size‐selected to an appropriate length for sequencing (typically 300–500 bp, average of ~380 bp). Thereafter, fragments were ligated to a Y‐adapter (P2), which ensures that all amplified fragments have the P1 and barcode, followed by the partial restriction site, a few bases of flanking sequence, and a P2 adapter (Davey & Blaxter, 2010). Finally, the DNA fragments representing a much‐reduced part of the original genome were PCR amplified using P1 and P2 primers and the RAD‐seq libraries were sequenced on the Illumina HiSeq2000 platform applying single‐read (1 × 100 bp) sequencing. Each plate was processed separately, with all samples within a plate pooled into a single library and sequenced on one lane.

Raw reads obtained from 100 bp single‐end Illumina sequencing were assessed for sequence quality, AT/GC content, and duplicate or overrepresented sequences using FastQC v.0.11.5. After initial quality assessment, reads were filtered and detection of single nucleotide polymorphism (SNP) was performed in Stacks v.1.42 pipeline (Catchen, Amores, Hohenlohe, Cresko, & Postlethwait, 2011; Catchen, Hohenlohe, Bassham, Amores, & Cresko, 2013; Hohenlohe et al., 2012) using the modules *process_radtags*, *denovo_map.pl* and *populations*.

As a reference genome was not available for *P. maculosus*, RAD tags were analyzed de novo, with parameters chosen according to the criteria showed in Paris et al. (2017). This was undertaken by clustering in loci similar sequence reads, at individual level, that had a maximum of two base pairs mismatches (*M* = 2) between them, and using only sequences with a minimum read depth of three (*m* = 3) were required to create a stack. After building of loci at the individual level, *cstacks* was used to match loci across samples and build a catalog, which allowed a maximum of one base pair mismatch (*n* = 1; Final coverage for each individual: mean = 57.77 X; stdev = 14.01). Subsequently, RAD tags for all individuals were used to detect only the first SNP (‐‐write_single_snp) by identifying at the same locus a marker that was present in one set of individuals but absent in another.

### 2.4 Filtering procedures

A second filtering step was performed in Plink 1.9. Individuals with more than 10% missing genotypes were excluded and only SNPs with a 90% genotyping rate (10% missing) and a minor allele frequency (MAF) higher than 5% were included. We also filtered data in respect to linkage among SNPs using a window size of 100 bp and a pairwise *r* ^2^ threshold of 0.2. Finally, markers that did not meet the Hardy–Weinberg Equilibrium (HWE) assumptions were excluded.

### 2.5 Summary statistics

We used the *F*_ST_ statistic (Wright, 1950) in order to relate the amount of genetic variation between populations from different sampling sites to the total genetic variation across populations (Meirmans & Hedrick, 2011). These indexes, as well as the expected heterozygosity (He) within each population, were obtained in GenoDive v.2.0.

### 2.6 Isolation‐by‐distance and isolation‐by‐environment

Correlation between genetic divergence and geographic distances was tested for IBD by applying the Mantel test to the linearized *F*_ST_ [*F*_ST_/(1 − *F*_ST_)] values and geographic distances (in kilometers). Geographic distances between sampling locations (i.e., the minimum distance between the locations by sea) were determined in Google Earth Pro v.7.3, and the Mantel test was executed in GenoDive v.2.0.

To test for IBE, we acquired geographic information systems (GIS) data for a total of nine water environmental variables (temperature, salinity, nitrate, phosphate, silicate, dissolved oxygen, chlorophyll, phytoplankton and primary productivity) from Bio‐ORACLE (Assis et al., 2018), and in addition, substratum rugosity was obtained from the QGIS terrain ruggedness index. The environmental matrix was filled out with values extracted for each variable at every location where tissue samples were collected. This information was centered and scaled before performing a principal components analysis (PCA) in R. All procedures were conducted using the R statistical software (R Development Core Team, 2017), by means of the following packages: *raster* (Hijmans, 2017), *rasterVis* (Lamigueiro & Hijmans, 2018), *maptools* (Bivand & Lewin‐Koh, 2015), *gridExtra* (Auguie, 2017), *lattice* (Deepayan, 2008) and *fields* (Nychka et al., 2017). A Mantel test was then performed in GenoDive v.2.0 to measure the linear relationship between environmental distance, as defined by the Euclidian distances in the PCA, and genetic distance, as measured by the *F*_ST_ statistic.

### 2.7 Distance‐based method (PCA)

We performed a PCA with the EIGENSOFT v.6.1.3 program in order to identify patterns in the data, by highlighting similarities and differences between samples and sampling sites.

### 2.8 Model‐based clustering analyses

The most probable number of genetic clusters and the membership of each individual to these clusters were estimated using the ADMIXTURE software (Alexander, Novembre, & Lange, 2009). The most likely number of clusters was selected based on cross‐validation error (CV) and the value of *K* that minimized the residuals (Pritchard et al., 2000). The prior expectation for the possible range of K (between 1 and 5) was based on the number of regions from which the samples were collected, that is the Arabian Gulf, the Sea of Oman, the Gulf of Aden, the Red Sea and Kenya.

### 2.9 Maximum likelihood evolutionary tree

To further investigate historical relationships and patterns of gene flow between populations, we used the TreeMix program (version 1.13; Pickrell & Pritchard, 2012), which provides a graphical representation of both population splits and migration events. TreeMix exhibits a branch‐and‐leaf structure, where the leaves represent populations and the branches are the inferred relationships between them. Additionally, the tree can account for situations where more than one branch may lead to the same leaf, suggesting population admixture and migration between populations.

Assuming the independence of SNPs (‐k 0), we ran TreeMix using the five sampled regions, and rooting the tree at the Red Sea, a suggested glacial refuge for many fish species during the last glaciation (DiBattista et al., 2013; DiBattista, Choat, et al., 2016). First, we assessed the tree topology with no migration events, which corresponded well with the ADMIXTURE and PCA results, and then we sequentially allowed for one to three migration events, performing 100 independent replications for each of them using the bootstrap option. The log‐likelihood value of each model was compared pairwise with the following model using the likelihood ratio test (LRT). The best model was selected if a significant difference was found between two consecutive models, and the corresponding residuals were visualized with the in‐built R script plotting functions in TreeMix v.1.13. The amount of variance in the relatedness between populations explained by the model was calculated using the R script *treemixVarianceExplained* (Card, 2015).

## 3 RESULTS

### 3.1 RAD‐seq summary

The RAD library of 151 specimens yielded a total of 787,286,606 reads, of which 633,985,956 remained after quality filtering and demultiplexing. These filtered reads were then used to create a catalogue containing 1,297,018 putative SNPs for construction of genotypes for all individuals. After the first filtering steps, Stacks yielded 100,275 SNPs. Further filtering procedures in Plink, such as missing genotype rate, minor allele frequency, linkage disequilibrium and nonconformance with Hardy–Weinberg Equilibrium, excluded one individual from the Arabian Gulf due to missing genotype data. A total of 10,225 genome‐wide SNPs from 150 individuals were retained and used in all subsequent analysis.

### 3.2 Summary statistics

Pairwise comparison between regions yielded values of *F*_ST_ ranging between 0.002 and 0.143 (*p* = .001) with the highest values observed in comparisons between the Arabian Gulf and the Kenyan coast, while the lowest values occurred between the Red Sea and the Gulf of Aden. Grouping the samples into three populations based on the clustering analyses (see ADMIXTURE and PCA results below), *F*_ST_ ranged from 0.043 between the Eastern Arabian Peninsula (EAP) and the Western Arabian Peninsula (WAP) populations, to 0.135 between Kenya and the EAP (*p* = .001; Tables 2 and 3).

**Table 2:** Pairwise *F*_ST_ values (below diagonal) for *P. maculosus* based on 10,225 SNPs

	Arabian Gulf	Sea of Oman	Gulf of Aden	Red Sea	Eastern Africa
Arabian Gulf	–	0.001	0.001	0.001	0.001
Sea of Oman	0.015	–	0.001	0.001	0.001
Gulf of Aden	0.052	0.033	–	0.001	0.001
Red Sea	0.049	0.032	0.002	–	0.001
Eastern Africa	0.143	0.130	0.112	0.103	–

Note

Significance *p*‐values are showed in the above diagonal.

**Table 3:** Pairwise *F*_ST_ values (below diagonal) for *P. maculosus* based on 10,225 SNPs

	EAP	WAP	EA
EAP	–	0.001	0.001
WAP	0.043	–	0.001
EA	0.135	0.102	–

Note

Significance *p*‐values are showed in the above diagonal.

Abbreviations: EA, Eastern Africa; EAP, Eastern Arabian Peninsula; WAP, Western Arabian Peninsula.

### 3.3 Isolation‐by‐distance and Isolation‐by‐environment

**Figure 2:**
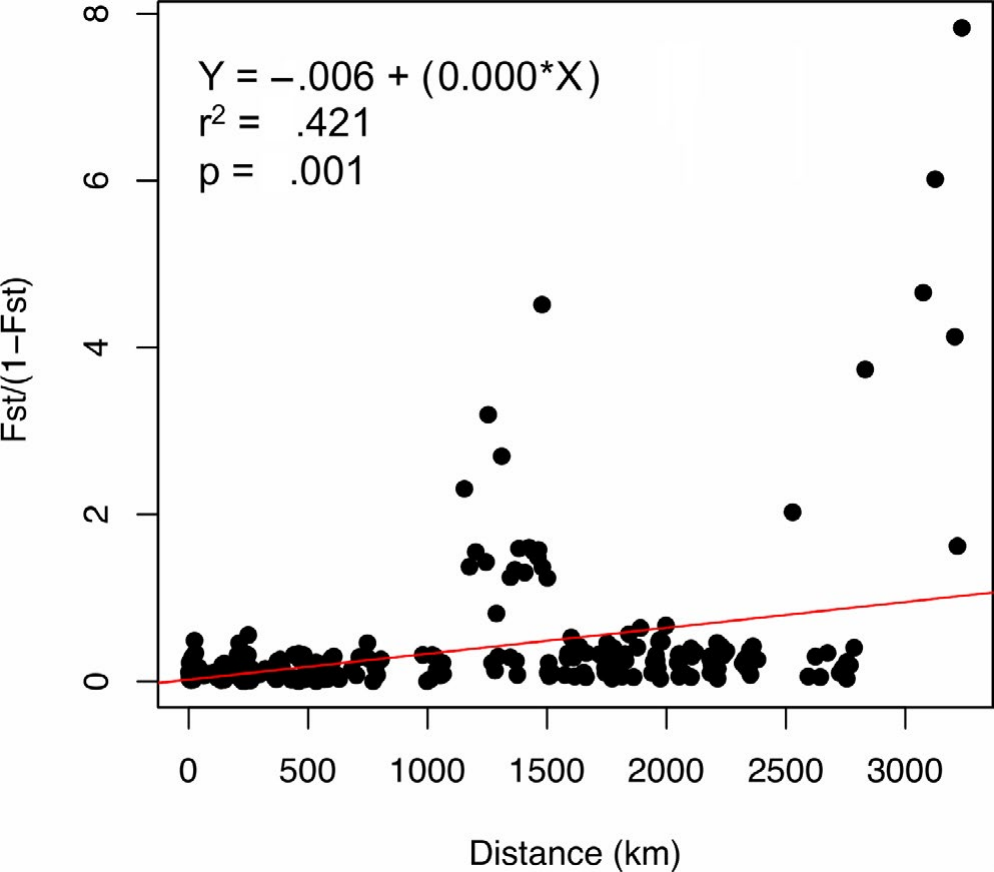
Relationship between pairwise geographic distance and genetic differentiation estimates (*F*_ST_/[1 − *F*_ST_]) for *Pomacathus maculosus* in the NIO

**Figure 3:**
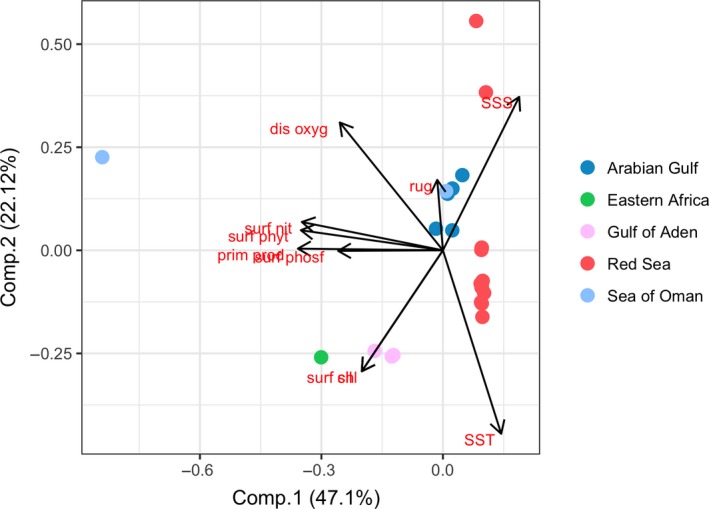
Principal component analysis based on the 10 environmental variables, dots represent the sampling sites where tissue samples were collected. chl, Chlorophyll; dis oxyg, dissolved oxygen; prim prod, primary productivity; rug, rugosity; SSS, sea surface salinity; SST, sea surface temperature; suf nit, surface nitrate; suf phyt, surface phytoplankton; surf phosf, surface phosphate; surf sil, surface_silicate

Geographic distances between sampling locations were significantly correlated (*r* ^2^ = .421, *p* = .001) with pairwise genetic distances (Figure 2). In contrast, no significant linear relationship was found between genetic and environmental distances (*r* ^2^ = .019, *p* = .523; Figure 3).

### 3.4 Historical population parameters

**Figure 4:**
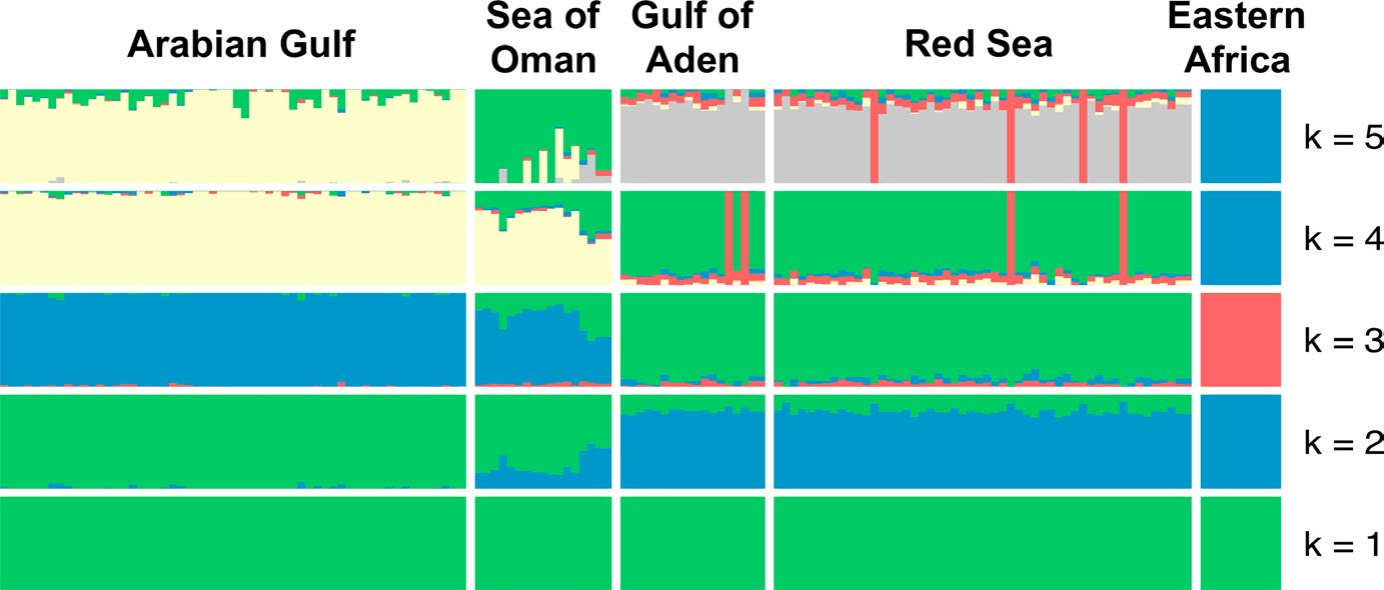
Population structure estimated by Admixture analysis. The 150 sampled individuals are represented by the vertical lines, which are partitioned into *K* colored segments that represent the individual's estimated membership fractions in *K* clusters. 10,225 SNPs

The Admixture analysis indicated the presence of population structure within the dataset. Cross‐validation error (CV) pointed to two values of *K* as the most likely number of clusters for *P. maculosus* (Figure 4). At *K* = 2 (CV = 0.56700), the clusters were comprised by a Western (the Red Sea, the Gulf of Aden and Kenya) and an Eastern (the Arabian Gulf and the Sea of Oman) cluster. At *K* = 3 (CV = 0.57138), individuals from Kenya composed a distinct group, and therefore suggested a division into three distinct clusters; a Western Arabian Peninsula cluster (Red Sea and Gulf of Aden), an Eastern Arabian Peninsula cluster (Arabian Gulf and Sea of Oman), and an eastern Kenya cluster. Although the Sea of Oman consistently clustered with the Arabian Gulf, this area is a very clear admixture zone (Figure 4).

### 3.5 Principal component analyses

**Figure 5:**
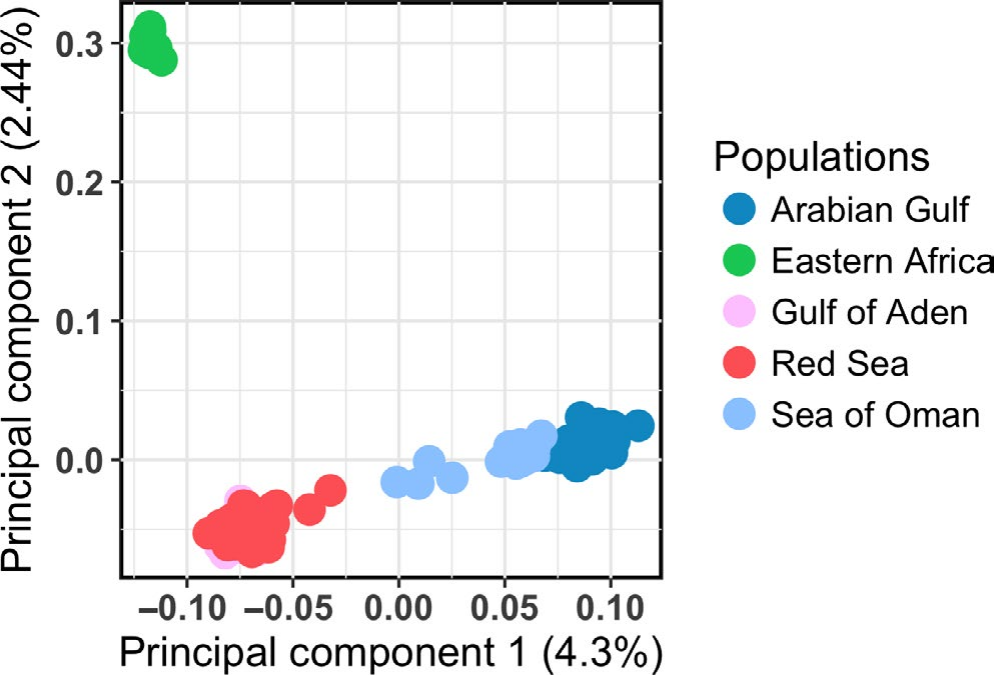
Principal component analysis of multilocus genotyped for 150 individuals of *Pomacanthus maculosus* from the NIO. The color scheme reflects geographic regions. 10,225 SNPs

The top two principal components (PCs) explained 6.74% of the total of genotypic variation. PC1 (4.3% of the variation) distinguished individuals sampled on the east side of the Arabian Peninsula from those obtained on the west side of the peninsula. Whereas PC2 (2.44% of the variation), to a higher extent, discriminated between individuals sampled off the coast of Kenya and those collected around the peninsula. Thus, one cluster included individuals from the western Arabian Peninsula (i.e., the Red Sea and the Gulf of Aden), the second cluster comprised eastern sampling sites (Sea of Oman and Arabian Gulf), and the third cluster consisted only of individuals from the African coast off Kenya (Figure 5).

### 3.6 TreeMix

**Figure 6:**
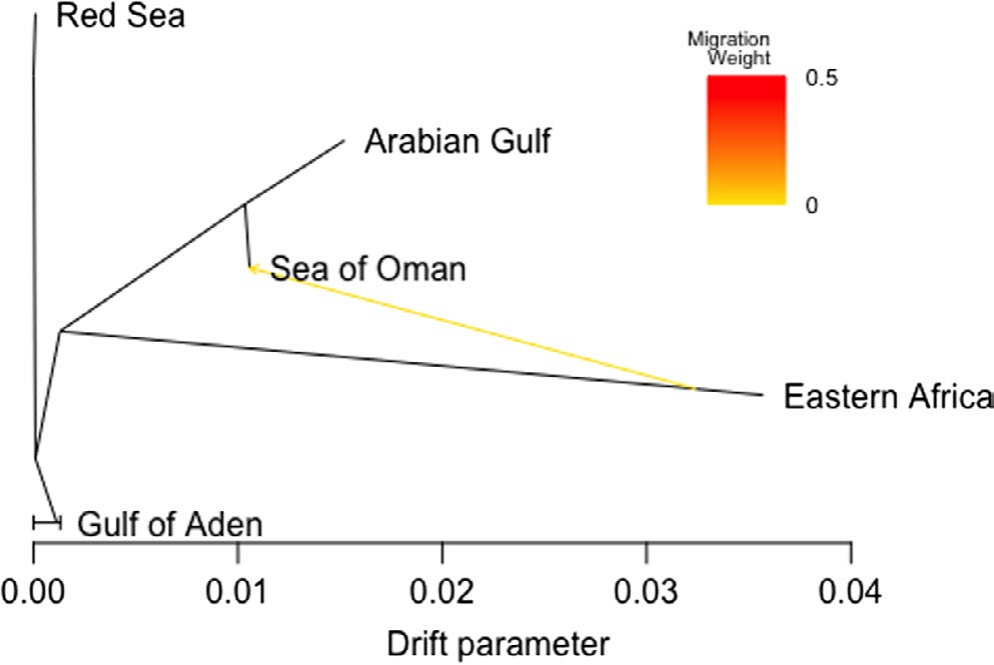
Maximum likelihood tree inferred by TREEMIX with the arrow indicating the migration event, the color represents its weight. 10,225 SNPs

The tree topology from TreeMix corresponded well with the ADMIXTURE and PCA results (Figure 6). The Red Sea and the Gulf of Aden clustered together in one branch, whereas the Arabian Gulf and the Sea of Oman clustered in another branch, and Kenya was placed in a third branch with a long drift parameter. The LRT showed no significant difference between the assessed models when allowing for either one, two or three migration events. Therefore, the most parsimonious model with one migration edge was chosen. The selected model explained 99.92% of the variance and showed very weak migration (*w* = 0.006) from Kenya to the Sea of Oman (Figure 6).

## 4 DISCUSSION

This work suggests the presence of two barriers that structure the population of *P. maculosus* into three genetic clusters across the sampled area: a subpopulation on the Eastern African coast represented by the samples collected in Kenya, and one distinct subpopulation on each side of the Arabian Peninsula. Although there were some important exceptions, we demonstrate an overall concordance between previously proposed biogeographic provinces, as defined by the taxonomic composition of fishes, and population genetic clustering of *P. maculosus*.

### 4.1 Searching for causes of genetic structure

#### 4.1.1 IBD and IBE

Dispersal has important consequences on the spatial distribution of genetic variation (Puebla et al., 2009). In coral reef fish, genetic structure driven by IBD is commonly attributed as the origin of the differentiation among populations (Planes & Fauvelot, 2002). Our study, however, showed that at the scale of the whole sample range (i.e., 3,000 km), IBD explained a significant but modest portion of the overall genetic variation in yellowbar angelfish. The decrease of the slope of IBD at such large spatial scales may be due to several factors (Puebla et al., 2009), such as the greater importance of mutation at large spatial scales (Rousset, 1997) and nonequilibrium conditions between migration and genetic drift (Slatkin, 1993).

We also sought to understand how the environment affects the distribution of genetic variation over populations by testing for IBE, but the analysis showed no significant relationship. *Pomacanthus maculosus* is distributed around the entire Arabian Peninsula, with the edges of the distribution (the Red Sea and the Arabian Gulf) being environmentally similar to each other (Figure 3). However, the genetic data showed that individuals from the Red Sea and the Arabian Gulf represent two distinct populations, and, therefore, no linear relationship between genetic and environment was detected.

We tested only for IBD and IBE, as alternative approaches such as Isolation by Biophysical Connectivity (IBC) requires biological information (e.g., PLD, larval vertical distribution, spawning season) that are not available for the majority of species inhabiting the NIO. Biophysical models would become oversimplified without sufficient biological information, and will thus likely fail to represent the characters of a single target species (but see Foster et al., 2012; Truelove et al., 2017).

#### 4.1.2 Oceanographic barriers

A possible reason for the distribution of genetic diversity observed in this study is the direct impact of ocean circulation forces on larval recruitment, as modeled in the IBC approach. Assuming this hypothesis, it might be suggested that the short spawning period of *P. maculosus* in the southern Arabian Gulf (September and October; Grandcourt & Francis, 2010), combined with the large degree of seasonality in ocean circulation across the NIO, creates a window of oceanographic conditions through which the larvae are released into the prevailing currents. During September and October, strong and steady winds blowing along the shore induce offshore movement of the Ekman layer, with consequent depression of the sea surface height (SSH) in coastal areas. This is followed by vertical advection of cooler waters from deeper layers to the surface, especially in the region off Somalia (Bruce, 1979) and Oman (Elliott & Savidge, 1990; Vic et al., 2017). During this period, ichthyoplankton located within the Ekman layer therefore tend to be transported offshore into areas of unsuitable habitat for settlement (Lett et al., 2007).

Despite potential negative impacts on larval recruitment, ocean circulation could also favor connection between the subpopulations of *P. maculosus*. During summer, when *P. maculosus* (Grandcourt & Francis, 2010) and other marine fish species spawn (Claereboudt, McIlwain, Al‐Oufi, & Ambu‐Ali, 2005; McIlwain et al., 2006), the wind blows southwest and drives the prevailing clockwise circulation in the Arabian Sea (see Cutler & Swallow, 1984). This period of clockwise upper ocean circulation represents the best opportunity for northwards dispersal of larvae from east Africa (Kemp, 1998), and could explain the (very weak) migration event from Kenya to the Sea of Oman indicated in the TreeMix analysis.

#### 4.1.3 Seascape barriers

**Figure 7:**
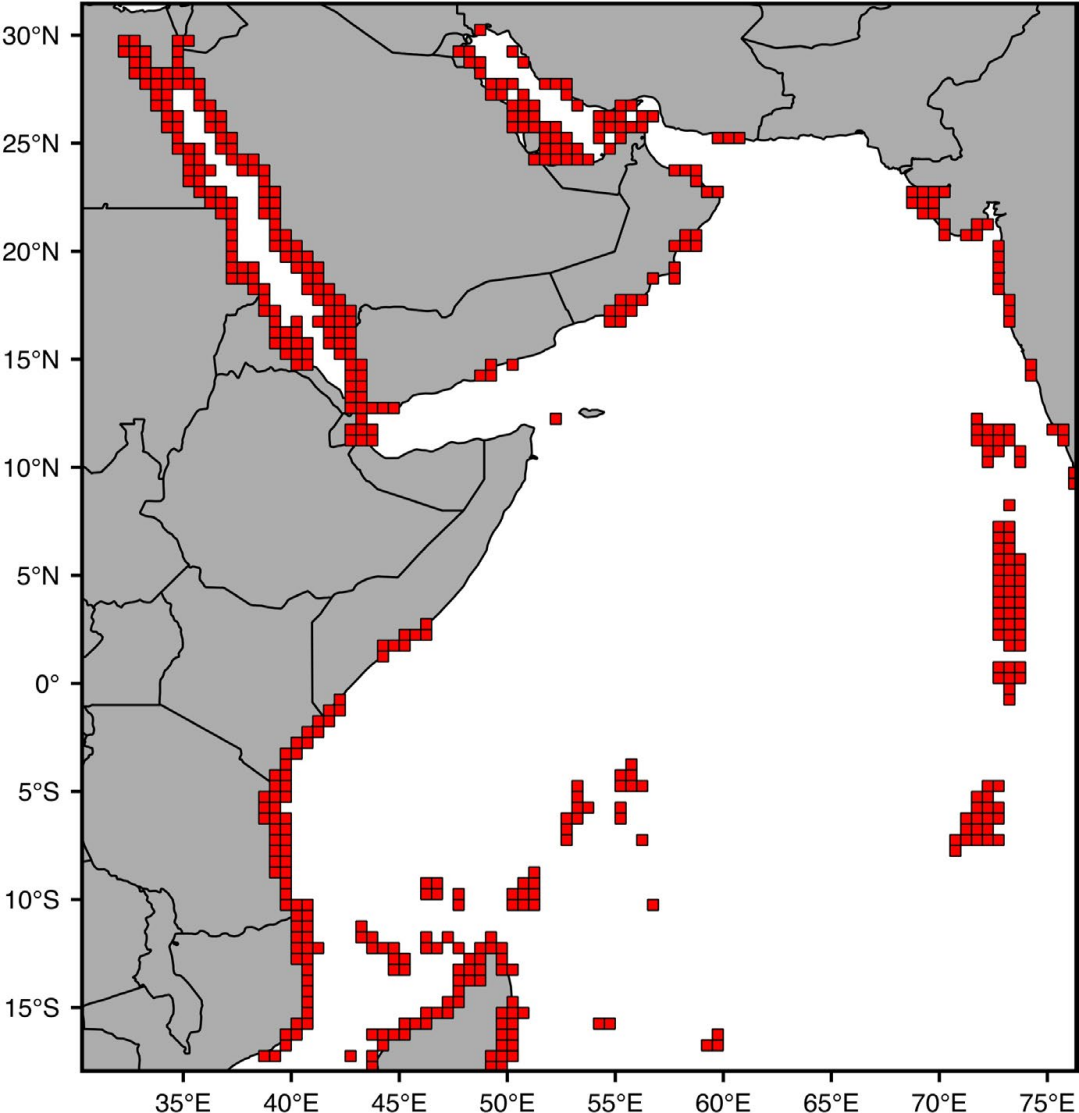
Map of the study area showing coral‐reef habitats available in the Western Indian Ocean (red squares)

An alternative hypothesis for the observed population genetic structure is that the lack of suitable habitats, in both the south of the Arabian Peninsula and along the Somali coast, creates an unbridgeable gap by restricting stepping‐stone connectivity between both the East and West side of the Arabian Peninsula and between the Arabian Peninsula and Kenya (Figure 7). According to this hypothesis, the seasonal upwelling events act as an indirect cause of genetic divergence by reducing the growth of suitable habitat (i.e., coral reefs) in these areas. For example, only four principal areas of reef coral occur along the Omani coast (Burt et al., 2016; Glynn, 1993), while a major break in habitat continuity occurs off the Somali coast where corals are reduced to patch reefs scattered within seagrass beds (Carbone & Accordi, 2000).

Besides the lack of suitable adult habitat between subpopulations, gene flow magnitude in fish (Rocha et al., 2002) and invertebrates (Ayre, Minchinton, & Perrin, 2009) may also vary due to differences in habitat specificities, such as rugosity. Coral reefs are topographically complex places that influence the associated organisms to various degrees either by increasing refuge from predators or by affecting the relationships between species (Gratwicke & Speight, 2005; Luckhurst & Luckhurst, 1978). To account for this hypothesis that habitat complexity affects the connectivity, our study considered the effect of rugosity on genetic structure in the IBE analysis, but this variable did not affect the relationship between genetic and environmental distance.

#### 4.1.4 Geological history

The endemism assigned to the NIO has also likely been augmented by geological history, as the seascape features of this area were drastically altered during the last glaciation events. This created barriers and changes in environmental conditions that likely led to the radiation of subpopulations or even species (DiBattista et al., 2013; DiBattista, Choat, et al., 2016; DiBattista, Roberts, et al., 2016; Klausewitz, 1972, 1989). For example, decreasing sea levels during glaciation caused significant alterations in environmental conditions by restricting water exchange between the Red Sea and Indian Ocean, and thereby creating a hypersaline environment within the Red Sea (DiBattista, Choat, et al., 2016; DiBattista, Roberts, et al., 2016). Also, the exposure of the seabed in the Gulf during this period (Vaughan et al., 2019; Lokier et al., 2015; Sarnthein, 1972) decreased the availability of habitat within the NIO. Therefore, the population currently inhabiting the Gulf is likely younger and tends to be genetically less diverse than neighboring populations (Hume et al., 2016). Indeed, our results show that the Eastern population (0.285) presented slightly lower heterozygosity compared to the Western population (0.293). Both populations showed higher heterozygosity than the African population (0.229), but this could potentially be explained by a smaller population size and/or by our restricted number of sampling sites along the African coast.

Although the radiation of *P. maculosus* is not clear to date, phylogenetic reconstruction carried out with thirteen *Pomacanthus* species revealed strong evidence that speciation within the genus has likely been a consequence of historical vicariance events, such as the Terminal Tethyan Event and the rise of the Isthmus of Panama (Hodge et al., 2013). Molecular analyses suggest that the speciation of *P. maculosus* occurred ~5 Mya (Hodge et al., 2013), a period that coincides with the origin of most reef fish species endemic to the Red Sea (Hodge et al., 2014). Nevertheless, the wide distribution of *P. maculosus* within the NIO has also led to the hypothesis that the species originated earlier, in the Mediterranean Tethys during the Pre‐Pliocene (Klausewitz, 1972).

### 4.2 Genetic population structure within the Western Indian Ocean (WIO)

Recent population genetic studies carried out within the WIO have supported a common geographic position of putative barriers across the Arabian Peninsula. The presence of a genetic discontinuity between both sides of the Arabian Peninsula as shown here for *P. maculosus*, coincides with previous population genetic reports from both mitochondrial DNA of *Cephalopholis hemistiktos* (Priest et al., 2016) and from double digest RAD sequencing (ddRAD‐seq) of *Amphiprion bicinctus* and *A. omanesis* (Saenz‐Agudelo et al., 2015), which in turn also presented a genetic break within the Red Sea (Nanninga et al., 2014). In contrast, mitochondrial DNA from a multi‐taxon survey around the peninsula revealed no evidence of population structure in nine of eleven fish species within the WIO. The two exceptions, *Chaetodon melannotus* and *Lutjanus kasmira*, were genetically structured by two different barriers, one between Oman and Socotra and one between Djibouti and Somalia, respectively (DiBattista et al., 2017). With respect to the genetic barrier off Somalia, five of the seven species examined in DiBattista et al. (2013) were genetically differentiated between the Red Sea and the WIO.

## 5 CONCLUSION AND FUTURE RESEARCH DIRECTIONS

There seems to be no single explanation or vicariance event that shaped the evolutionary histories of fish species within the NIO. Therefore, comparative phylogeography studies could represent an initial endeavor to detect and measure the relative importance of the major evolutionary forces preventing the gene flow and shaping the patterns of genetic diversity. Moreover, future genetic work, particularly studies using advanced genomic approaches (e.g., whole genome sequencing) could provide greater resolution for particular taxa of interest. On the other hand, biogeographic studies in the WIO are still hindered by socioeconomic and political restraints in some of the countries bordering the region, which have created a situation of limited access for scientists. For example, the sea off Somalia coincides with the major faunal change between the Arabian Peninsula and the WIO, but is largely unstudied, much like the understudied region off the coast of the Arabian Sea between the Gulf of Aden and southern Oman. These areas are not well characterized, and not solely in terms of species distributions, but also in terms of habitats.

## CONFLICT OF INTEREST

None declared.

## AUTHOR CONTRIBUTIONS

FT, PR, RB and PRM conceived the ideas; all authors conducted the fieldwork and collected the data: Red Sea (MLD, JDD); Gulf of Aden (MLD, JDD); Sea of Oman (AM); Qatar (RR, PR, PFT, EES, PRM); Abu Dhabi (DDA, DF, JB); Kenya (PRM, FT). FT conducted laboratory work and performed statistical analysis. FT led the writing with assistance from all authors; all authors contributed to revisions of the manuscript.

## Data Availability

The genetic data reported in this paper have been deposited in the National Center for Biotechnology Information (NCBI) Short Read Archive under the following BioProject Accession Numbers: PRJNA559342 (*Pomacanthus maculosus* Raw sequence reads).
